# Comorbidity, disability, and healthcare expenditure of ankylosing spondylitis in Korea: A population-based study

**DOI:** 10.1371/journal.pone.0192524

**Published:** 2018-02-08

**Authors:** Jeong Seok Lee, Baek-Lok Oh, Hee Young Lee, Yeong Wook Song, Eun Young Lee

**Affiliations:** 1 Division of Rheumatology, Department of Internal Medicine, Seoul National University College of Medicine, Seoul, Korea; 2 Department of Ophthalmology, Seoul National University College of Medicine, Seoul, Korea; 3 Center for Preventive Medicine and Public Health, Seoul National University Bundang Hospital, Seongnam, Gyeonggi-do, Korea; University of Oxford, UNITED KINGDOM

## Abstract

**Background:**

Ankylosing spondylitis (AS) is an inflammatory rheumatic disease typically diagnosed in young age and follows a chronic progressive course. Its impact on the patient is life-long and the burden that AS exerts on society is increasing cumulatively every year. We aimed to quantify the burden of AS and to identify the factors associated with comorbidity, disability, and healthcare expenditure in Korean AS patients.

**Methods:**

We conducted a nationwide, population-based study using health insurance data (2003–2013). The analysis included individuals with incident AS (1,111 patients) and controls (5,555 patients) matched by age, sex, income, and geographic region. The incidence rates of extra-articular manifestations (EAMs), comorbidities, mortality, and disability (type and severity) were compared between AS patients and controls. Annual health expenditure per patient was also analyzed. Associations were expressed as odds ratios (ORs) with 95% confidence intervals (95%CIs).

**Results:**

During the follow-up, 28% of AS patients experienced at least one EAM. AS diagnosis was significantly associated with Charlson comorbidity index ≥3 (OR 2.18, 95% CI 1.91–2.48). Disability rate was higher in AS patients than in controls regardless of cause and severity (OR 2.94, 95% CI 2.48–3.48), but crude incidence rate ratios for mortality were not significantly higher. On multivariate analysis, male sex (OR 3.18, 95% CI 2.13–4.75), presence of an EAM (OR 1.63, 95% CI 1.15–2.32), and older age at diagnosis (OR 1.27, 95% CI 1.20–1.35) were evidently associated with increased disability in AS. Presence of an EAM was also associated with increased AS-unrelated expenditures in biologic-naïve patients (median, 1112 vs. 877 USD per person, *P* < 0.05).

**Conclusions:**

In patients with AS, demographic factors and systemic manifestations including EAMs and other comorbidities were associated with increased disability and healthcare expenditures.

## Introduction

Ankylosing spondylitis (AS) is an inflammatory rheumatic disease with musculoskeletal and systemic manifestations [1]. Because AS is typically diagnosed before the age of 40 years and follows a chronic progressive course [2], its impact on the patient is life-long. In addition to the burden on the individual patient, the burden that AS exerts on society is increasing cumulatively every year [3]. This burden is not confined to healthcare costs associated with alleviating back pain and stiffness caused by the disease itself [4–6], but also includes costs associated with managing extra-articular manifestations (EAMs), comorbidities, disability, and mortality, to which AS contributes indirectly [7].

EAMs and comorbidities represent both manifestations and consequences of AS. The close association between AS and EAMs such as uveitis, inflammatory bowel disease (IBD), and psoriasis was revealed by clinical observation during the 1960s [8, 9]. Additionally, common comorbidities including osteoporosis, vertebral fracture, and cardiovascular disease exacerbate the burden of AS patients [10–12]. The outcomes of recent population-based studies evaluating the incidence and severity of comorbidities and EAMs in AS suggest it is difficult to describe the true burden of AS, and that a more comprehensive investigation approach is required [13–15].

While AS is not usually described as a fatal disease, recent data from a large-scale, nationwide, cohort study indicated significantly higher mortality rates in AS [16, 17]. In terms of disability, AS-related pain had a strong and detrimental effect on the sense of well-being, resulting in reduced work capability and employment status [18, 19]. Although mortality and disability are the typical outcomes evaluated for chronic diseases, such outcomes were investigated to a relatively lower extent in AS than in rheumatoid arthritis (RA) [2, 20–23]. Overall, the per-patient healthcare costs in AS seem to be lower than those in RA [4, 5]. However, the exact clinical factors associated with higher costs remain unclear, as the healthcare costs of both diseases seemed to depend largely on whether or not biologic agents are used for the treatment [24]. We previously assessed the cost of illness in Korean patients with AS, but the implications of our findings were inevitably limited because we used data from a single center [25].

To quantify the burden of AS in terms of disability, mortality, and healthcare costs in Korea, we conducted a nationwide, population-based study of national health insurance data.

## Materials and methods

### Study design

This was a population-based, nested case-control study of patients with AS and matched controls. Disability, mortality, and healthcare costs were investigated as primary outcomes. The study was exempt from ethical review by the institutional review board because the data originated from de-identified secondary data released by the National Health Insurance Service for research purposes. For the same reason, patient consent was not required for the present analysis. All individuals included in the National Sample Cohort provided informed consent for data collection, storage, and processing.

### Data sources

Our study was based on the National Health Insurance Service-National Sample Cohort (NHIS-NSC), which is a Korean population-based cohort of individuals who submitted medical care claims between 2002 and 2013 [26]. The cohort, which consisted of 1,025,340 individuals, was obtained by age-, sex-, and income-based stratification sampling of approximately 50 million Koreans registered with the Korean NHIS in 2002. Since the NHIS-NSC was obtained based on a data set containing de-identified secondary data released for research purposes, the present study was exempt from ethical review by the institutional review board.

### Selection of patients and controls

Patients diagnosed as having AS were identified based on the diagnosis code M45 according to the 10th version of the International Classification of Diseases (ICD-10). We further considered only patients who were diagnosed in a general hospital and were older than 15 years at initial diagnosis (S1 Fig). After excluding 88 patients who had been diagnosed as having RA (ICD-10 code: M05, M06) or systemic lupus erythematosus (ICD-10 code: M32), 1,372 patients with prevalent AS were further considered. The first year (2002) was considered a washout period, and patients diagnosed during this time were excluded. Finally, 1,111 patients with incident AS (i.e., diagnosed between 2003 and 2013) were selected from the NHIS-NSC as the study group. Additionally, 5,555 controls (five controls per each AS patient) were selected from among individuals insured between 2002 and 2013 and who were not diagnosed as having AS, RA, or systemic lupus erythematosus. We did not exclude the population with other diseases when selecting control group. The controls were randomly matched to the study group by age group (4 strata), sex (2 strata), income level (5 strata), and geographic region (3 strata) at diagnosis (S1 Table).

### Study variables

Age at diagnosis, sex, duration of follow-up, geographic region, type of social security, and household income were compared among all AS patients, a subgroup of AS patients treated with biologic agents, and controls. Co-occurrence of EAMs including uveitis (ICD-10 codes: H20 and H22), psoriasis (L40), and IBD (K50 and K51) was noted. Subgroups with higher risk of EAMs such as biologic agent users, male, and age<45 years were selected for evaluating pattern of EAM prevalence. The Charlson comorbidity index (CCI) was also calculated for each patient [27].

Annual healthcare expenditure, disability, and mortality rates were included as dependent variables. Annual healthcare expenditure covered by national health insurance of Korea includes medical costs for diagnostic test, treatment including surgery, intervention or medication, and outpatient or inpatient care. As most of medical service for caring AS are covered by national insurance, this annual healthcare expenditure is almost equal to direct medical cost. AS-related and unrelated costs were counted separately based on the presence of the diagnosis code for AS. In terms of disability, the category (physical disability or all-cause disability) and severity (grade 1 or 2, severe; grade 3–6, mild) were provided from NHIS-NSC which were evaluated by responsible physician according to the specific guidelines established by Korean government. Regarding physical disability, we presented specific criteria for defining spinal deformity including AS as a example (S1 Text). However, specific subclass of physical disability such as spinal defomity was not provided in this database.

### Statistical analysis

The sociodemographic features of the study group (patients with AS) and matched controls are presented in terms of number of patients and prevalence. Odds ratios (ORs) with 95% confidence intervals (95%CIs) were analyzed for the relationship between each variable and each outcome, with the alpha level set at *P* < 0.05. Disability and mortality rates were calculated per 1000 person-years and used to calculate incidence rate ratios (IRRs; with 95%CIs) per 1000 person-years at risk, evaluating the risk for a certain outcome in the study group compared to the corresponding risk in the control cohort. Multivariate unconditional logistic regression analysis was also performed to investigate the risk factors of disability and mortality in AS patients. The median and interquartile range (IQR) of annual health expenditures were calculated and compared between the study group and the control cohort using Mann-Whitney U test. The SAS Enterprise Guide (version 5.1, SAS Institute Inc., Cary, NC, USA) was used to perform the analysis.

## Results

### Demographic and clinical characteristics of the study group and control cohort at diagnosis

The sociodemographic characteristics of the study group (1,111 patients with AS) and control cohort (5,555 patients without AS) at diagnosis are shown in Table 1. The demographics (sex, age group, household income, and geographic region at initial diagnosis) were similar between the study group and the control cohort, indicating appropriate matching. The subgroup analysis involved 128 AS patients treated with biologic agents, who were relatively younger and more likely to be male. Neither the income level nor the type of social security was associated with the use of biologic agents.

**Table 1:** Sociodemographic characteristics of the study group (patients with AS) and matched controls.

Characteristic	AS patients (n = 1111)	AS patients treated with biologics (n = 128)	Controls (n = 5555)
**Sex**			
Male (%)	725 (65)	108 (84)	3625 (65)
**Age at diagnosis**			
15–29 years	315 (28)	41 (32)	1575 (28)
30–44 years	366 (33)	57 (45)	1830 (33)
45–59 years	258 (23)	26 (20)	1290 (23)
≥60 years	172 (16)	4 (3)	860 (16)
**Duration of follow-up**			
<5 years	423 (38)	48 (38)	2115 (38)
≥5 years	688 (62)	80 (62)	3440 (62)
**Geographic region**			
Seoul, metropolitan	247 (22)	35 (27)	1235 (22)
Large cities^a^	225 (20)	29 (23)	1125 (20)
Other areas	639 (58)	64 (50)	3195 (58)
**Type of social security**^**b**^			
Health insurance	1077 (97)	119 (93)	5385 (97)
Medical aid	34 (3)	9 (7)	170 (3)
**Household income**			
1st quintile	146 (13)	25 (20)	730 (13)
2nd quintile	160 (14)	23 (18)	800 (14)
3rd quintile	206 (19)	17 (13)	1030 (19)
4th quintile	254 (23)	29 (23)	1270 (23)
5th quintile, highest	345 (31)	34 (26)	1725 (31)

Data given as number of patients (percentage). Controls were matched to the study group by age, sex, income, and geographic region.

^a^ Included six major cities (Busan, Daegu, Incheon, Gwangju, Daejeon, and Ulsan) in Korea.

^b^ Type of social security at diagnosis was determined according to the household income level, with the 5^th^ quintile indicating the highest income.

AS, ankylosing spondylitis.

### EAMs and comorbidities

**Fig 1:**
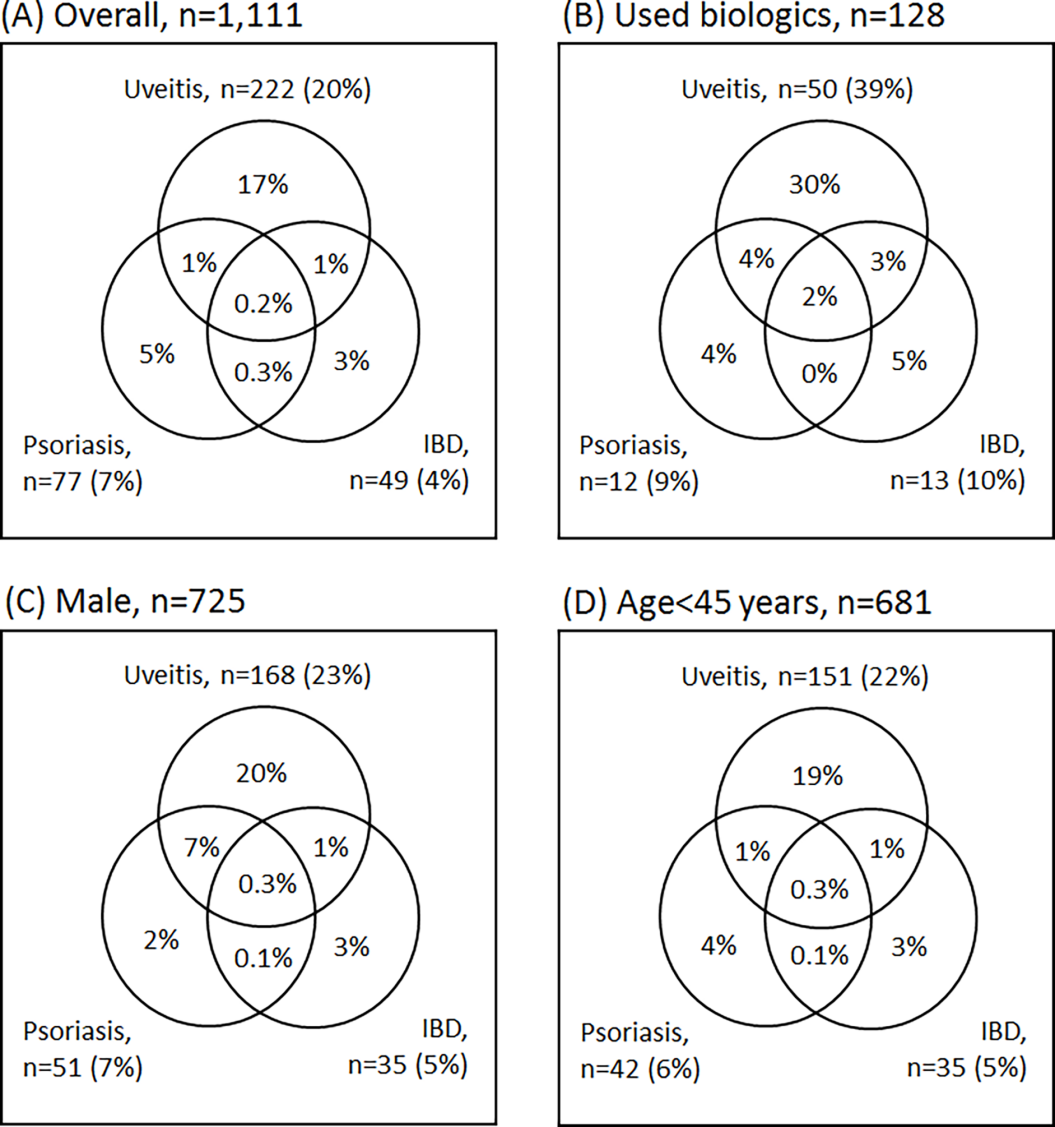
Co-occurrence of extra-articular manifestations in patients with ankylosing spondylitis. IBD, inflammatory bowel disease.

Uveitis was the most prevalent EAM (20%), and occurred 9 times more frequently in AS patients than in controls (*P* < 0.001) (Table 2). In general, 28% of AS patients experienced at least one type of EAM (OR, 5.25; 95%CI, 4.44–6.20), and 3% of AS patients had multiple EAMs (OR, 10.95; 95%CI, 5.91–20.30). Male patients (32%) and patients treated with biologic agents (48%) tended to have more EAMs than the other AS patients, especially in combination with uveitis (Fig 1).

**Table 2:** Extra-articular manifestations (EAMs) in patients with ankylosing spondylitis (AS) and in controls.

Type of EAM^a^	Number of patients (%)		Odds ratio (95%CI)	P-value
	AS patients (n = 1111)	Controls (n = 5555)		
At least one EAM	314 (28)	388 (7)	5.25 (4.44–6.20)	<0.001
Uveitis	222 (20)	150 (3)	9.00 (7.23–11.20)	<0.001
Psoriasis	77 (7)	185 (3)	2.16 (1.64–2.84)	<0.001
IBD	49 (4)	69 (1)	3.67 (2.53–5.32)	<0.001
Two or more EAMs	32 (3)	15 (0.3)	10.95 (5.91–20.30)	<0.001

Both the study group (AS patients) and the control cohort were selected from the National Health Insurance Service-National Sample Cohort (NHIS-NSC). Controls were matched to the study group by age, sex, income, and geographic region.

^a^ Based on appropriate International Classification of Disease codes listed in the NHIS-NSC claims.

95%CI, 95% confidence interval; IBD, inflammatory bowel disease.

AS patients had a significantly higher incidence of CCI ≥ 3 (OR, 2.18; 95%CI, 1.91–2.48) (S2 Table). In the study group, male sex, younger age at diagnosis (<45 years old), and presence of an EAM were associated with increased risk of higher CCI relative to the risk noted in the control cohort.

### Disability and mortality rates

Crude incidence rates of disability in AS patients and the control cohort were calculated (S3 Table). Compared to controls, patients with AS had higher disability rates, regardless of cause and severity (OR, 2.94; 95%CI, 2.48–3.48) (Table 3). The incidence of physical disabilities was much higher in AS patients than in controls (OR, 5.33; 95%CI, 4.31–6.60), and the trend was even stronger for severe physical disability (OR, 11.47; 95%CI, 6.31–20.83). Regarding mortality, the crude incidence rates for the overall study group of AS patients were not significantly higher than those for controls (S4 Table).

**Table 3:** Incidence rate ratios comparing disability rates between the study group (AS patients) and the control cohort.

Risk factor	Incidence rate ratio (95%CI)^a^			
	All-cause	All-cause, severe	Physical	Physical, severe
**All patients**	2.94 (2.48–3.48)	2.12 (1.52–2.97)	5.33 (4.31–6.60)	11.47 (6.31–20.83)
**Sex**				
Male	3.45 (2.83–4.22)	2.49 (1.70–3.64)	6.80 (5.27–8.77)	13.29 (6.76–26.14)
Female	2.01 (1.45–2.80)	1.34 (0.64–2.77)	3.01 (2.00–4.52)	6.86 (1.79–24.88)
**Age at diagnosis**				
<45 years	2.64 (1.99–3.50)	1.23 (0.70–2.15)	5.40 (3.80–7.67)	8.64 (3.58–20.84)
≥45 years	3.30 (2.66–4.08)	3.32 (2.15–5.11)	5.58 (4.27–7.29)	15.03 (6.62–34.12)
**Household income**				
<4th quintile	2.80 (2.22–3.53)	2.09 (1.37–3.20)	5.64 (4.20–7.57)	13.94 (6.42–30.27)
≥4th quintile, high	3.15 (2.45–4.05)	2.23 (1.29–3.86)	5.06 (3.72–6.89)	8.57 (3.32–22.10)
**Duration of follow-up**				
<5 years	2.72 (2.27–3.25)	1.76 (1.25–2.48)	5.01 (3.99–6.30)	9.60 (5.20–17.72)
≥5 years	1.02 (0.55–1.88)	1.44 (0.31–6.79)	1.75 (0.86–3.54)	5.76 (0.36–92.16)
**EAM**				
≥1	3.49 (2.31–5.27)	3.55 (1.62–7.80)	9.11 (4.65–17.84)	11.99 (2.74–52.42)
None	2.57 (2.10–3.14)	1.63 (1.07–2.48)	4.60 (3.61–5.86)	8.84 (4.40–17.78)
**Comorbidity**				
CCI ≥ 3	2.19 (1.78–2.69)	1.97 (1.30–2.99)	3.62 (2.78–4.70)	10.68 (4.78–23.88)
CCI = 2	3.48 (2.24–5.41)	2.41 (0.98–5.91)	6.74 (3.91–11.60)	12.91 (2.50–66.54)
CCI = 1	2.58 (1.52–4.39)	2.25 (0.91–5.58)	5.15 (2.80–9.50)	9.85 (2.65–36.70)
CCI = 0	4.72 (2.36–9.45)	No mortality	15.06 (6.24–36.33)	No mortality

Both the study group (AS patients) and the control cohort were selected from the National Health Insurance Service-National Sample Cohort. Controls were matched to the study group by age, sex, income, and geographic region.

^a^per 1000 person-years.

95%CI, 95% confidence interval; AS, ankylosing spondylitis; CCI, Charlson comorbidity index; EAM, extra-articular manifestation.

**Fig 2:**
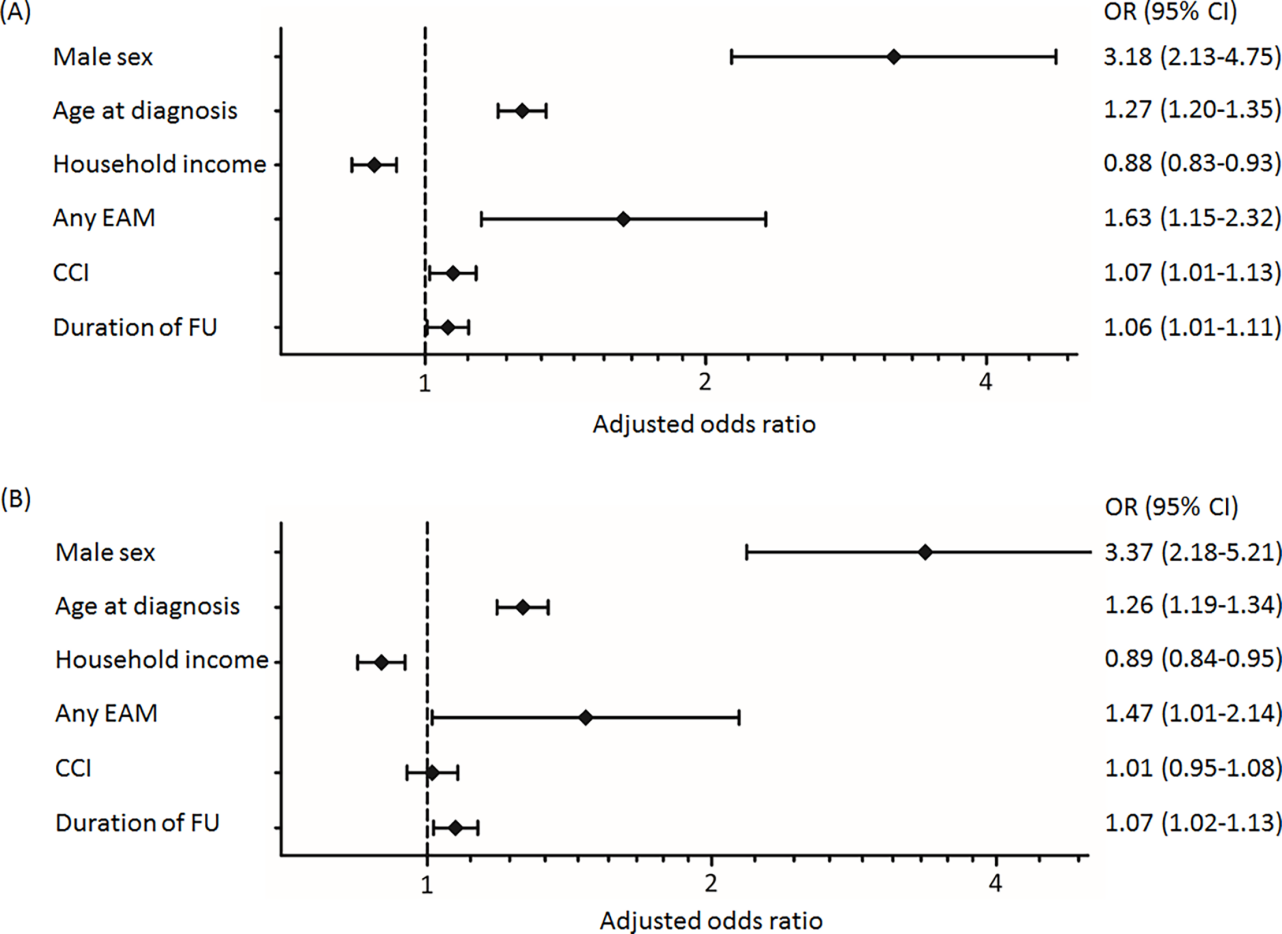
Forest plot presenting the result of multivariate logistic regression analysis for disability within the study group of patients with ankylosing spondylitis. (A) All-cause disability. (B) Physical disability. 95% CI, 95% confidence interval CCI, Charlson comorbidity index; EAM, extra-articular manifestation; FU, follow-up; OR, odds ratio.

On multivariate analysis, male sex (OR, 3.18; 95%CI, 2.13–4.75), presence of an EAM (OR, 1.63; 95%CI, 1.15–2.32), older age at diagnosis (OR, 1.27; 95%CI, 1.20–1.35), higher CCI score (OR, 1.07; 95%CI, 1.01–1.13), and longer duration of follow-up (OR, 1.06; 95%CI, 1.01–1.11) were risk factors associated with all-cause disability (Fig 2A). Higher income level (OR, 0.88; 95%CI, 0.83–0.93) was inversely correlated with disability. A similar pattern of association was observed regarding physical disability (Fig 2B) and severe disability (S2 Fig).

### Annual health expenditures

The annual health expenditures for patients with AS were 3.3 times higher than those for controls (median, 596 vs. 183 USD) (S5 Table). Because of the high cost of the medications, the expenditures for AS patients who were prescribed biologic agents were 24.9 times higher than those for controls (median, 4549 vs. 183 USD). Factors such as female sex, older age (≥45 years old), lower income level, shorter duration of follow-up, presence of EAMs, and higher CCI were significantly associated with increased annual health expenditures. Among individuals who experienced disability or death during the study period, AS patients had significantly higher annual health expenditures than those of controls.

**Fig 3:**
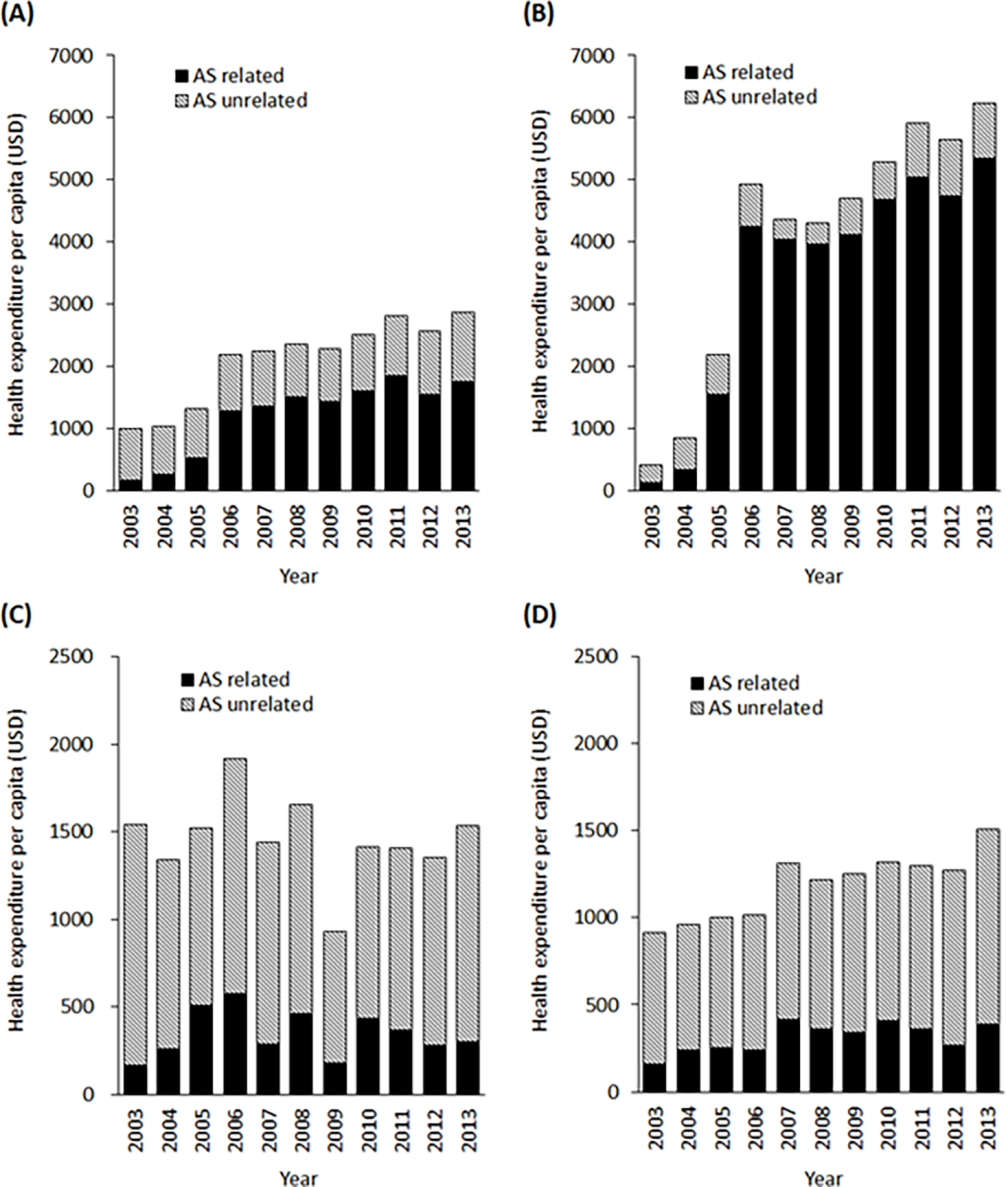
Health expenditure per capita in ankylosing spondylitis (AS). (A) All AS patients. (B) AS patients prescribed biologic agents at least once. (C) Biologic-naïve AS patients with EAMs. (D) Biologic-naïve AS patients without EAMs. EAM, extra-articular manifestation.

Analyzing the year-by-year expenditures for AS patients revealed that the cost per capita has increased significantly since 2006, which is when biologic agents began to be covered by the NHIS (Fig 3A). When splitting the annual cost based on the history of biologic agent prescription or the purpose of the expense (AS-related or AS-unrelated), we found that patients prescribed biologic agents had higher AS-related expenditures but lower AS-unrelated expenditures (median, 898 vs. 602 USD per person, *P* = 0.00026) (Fig 3B) compared to the values noted for biologic-naïve patients. On the other hand, in biologic-naïve patients, presence of an EAM was associated with increased AS-unrelated expenditures (median, 1112 vs. 877 USD per person, *P* = 0.0068), without significant effect on AS-related costs (median, 347 vs. 312 USD per person, *P* = 0.46) (Fig 3C and 3D).

## Discussion

This is the first study to quantitatively assess the burden of AS using representative claim data of the national health insurance service, which covers more than 95% of the Korean population. We found that the disability rate and annual health expenditures were significantly higher in the study group (patients with AS) than in the control cohort (patients without AS, matched with AS patients for sex, age group, income level, and geographic region). We found that the burden of AS was aggravated by clinical features including EAMs, as well as by demographic factors known to be related to AS, such as male sex, old age, and lower income.

The EAMs compounded the influence of comorbidities and increased the rate of disability and the healthcare costs of AS patients. The prevalence of EAMs (uveitis, 20%; psoriasis, 7%; IBD, 4%) in the patients with AS included in the present study was similar to the previously reported prevalence of such conditions (20–30%, 7–9%, and 1–6% for uveitis, psoriasis, and IBD, respectively) [7]. The presence of multiple EAMs is recognized as systemic involvement of AS and, if not effectively controlled using nonsteroidal anti-inflammatory drugs or disease modifying anti-rheumatic drugs, is believed to increase the need for biologics such as anti-tumor necrosis factor (TNF) agents. As expected, AS-related costs were higher in AS patients with EAMs who had been prescribed biologics at least once; however, the AS-unrelated costs were reduced in these patients compared to those in biologics-naïve patients with EAMs. A previous study also showed reduced rates of uveitis and IBD flares in patients treated with anti-TNF agents [28]. Taken together, these findings highlight the need for careful monitoring and active treatment of AS patients with EAMs.

Although the aspect of disability was evaluated in previous studies regarding the burden of AS, most such investigations analyzed work-related or patient-reported disabilities [18, 19]. In Korea, the government provides extended medical and welfare benefits depending on the cause and severity of the disability. Therefore, determining the cause and severity of disability is a complex process performed according to strict protocols by experienced physicians and government officers (S1 Text). Therefore, we believed that the data on disability for our cohort is objective and valuable, reflecting the real-world impact of the disease. For patients with AS, we found that comorbidities were not associated with the incidence of disability, which might be related to the fact that the CCI-based categories did not contain AS itself or related co-morbidities such as EAMs. On the other hand, AS had an impact on the rate of disability independently from other common comorbidities.

The impact of AS on mortality was recently highlighted in large-scale, population-based studies, according to which all-cause mortality (OR, 1.60; Swedish cohort) and cardiovascular mortality (OR, 1.36; Canadian cohort) were higher in patients with AS than in controls [16, 17]. Compared to the Swedish cohort in terms of median age, the AS patients in the present study group were relatively younger; moreover, the proportion of patients older than 60 years was 8% lower in our study group. We found that mortality was not significantly higher in the AS patients. Unlike mortality, several factors such as male sex, presence of an EAM, and older age at diagnosis were commonly associated with increased disability regardless of cause and severity. Even though EAMs might not exert adverse effects directly on the musculoskeletal system, presence of an EAM can be used as a prognostic factor indicating increased burden of physical disability. Advanced age at AS diagnosis was also associated with increased disability rates and higher health expenditures after adjustment for confounders, possibly suggesting poor prognosis for elderly patients who had delayed diagnosis or late onset of AS.

Annual health expenditures per patient are difficult to compare across different countries because of the high variation in healthcare policies. In absolute values, costs associated with the AS patients included in our cohort are lower (around 1600 USD per patient-year) than the values noted in most countries (e.g., Netherlands, around 6400 USD per patient-year; Sweden, around 5800 USD per patient-year) [29]. Moreover, in Korea, AS has been classified as a rare and intractable disorder, and 90% of AS-related healthcare costs are covered by the NHIS. Therefore, Korean patients with AS had a lower barrier to receive healthcare, including expensive biologic agents. Given our finding that biologic agents reduced AS-unrelated costs, and considering their possible effect on reducing indirect costs such as those related to work productivity, further study is warranted to evaluate cost-effectiveness and social impact.

The present study has several limitations. First, we had to include only patients who were diagnosed by the physicians of general hospitals in Korea. As the most common initial complaint of AS is back pain, primary physicians may also diagnose AS even when the evidence is not definitive. However, because of the low barrier to obtaining pricey healthcare in Korea, more than two thirds of NHIS expenditures for AS (ICD-10 code: M45.x) were spent in referral hospitals [3]. AS severity might be exaggerated in our cohort, but the accuracy of the AS diagnosis is likely high because most general hospitals have hired rheumatology specialists since the early 2000s [30].

Second, the intrinsic weakness of claims data could not be overcome. Because of strict regulations related to the diagnosis and registration of rare and severe diseases, the accuracy of diagnosing AS is very high in Korea. However, important clinical information about AS–such as disease activity (e.g., Bath Ankylosing Spondylitis Disease Activity Index), radiographic outcomes, human leukocyte antigen B27 (HLA-B27) status, and behavioral factors (i.e., smoking)–could not to be included in the analysis. Hence, we could not draw a conclusion with regard to the relevance of such factors in the context of disability, mortality, and healthcare costs. Recently established large-scale registries of AS, such as the registry of Korean patients with rheumatic disease using biologics (KOBIO, ClinicalTrials.gov Identifier: NCT01965132), may provide insight into the relationship among these factors and help develop active treatment strategies to reduce the burden of AS.

Third, the follow-up duration of the patients with AS was not consistent. Moreover, the maximum duration of observation was only 11 years, which might be too short to evaluate the true outcomes of AS considering its chronicity. The treatment strategy for AS changed after the introduction of biologic agents as a therapeutic option. To evaluate the effect of biologic agents on the disease burden of AS, long-term follow-up of the patients who were prescribed biologic agents after 2004 is warranted.

Despite its limitations, the present study showed that AS is not only a painful disease of the musculoskeletal system, but also has systemic consequences such as EAMs and other comorbidities, and that these factors are associated with increased disability rates and healthcare expenditures. Early diagnosis and active treatment for the patients having such risk factors might help reduce the burden of disease on the patients, on the health care system, and on society.

## Supporting information

S1 Fig Flow chart for selection of patients included in the final analysis. (DOCX)

S2 Fig Forest plot presenting the result of multivariate logistic regression analysis for severe disability within the study group of patients with ankylosing spondylosis. (DOCX)

S1 Table Stratification of demographic variables used for matching. (DOCX)

S2 Table Risk for overall comorbidity with a given Charlson comorbidity index (CCI) in ankylosing spondylitis. (DOCX)

S3 Table Crude incidence rates of disability in the study group (AS patients) and the control cohort. (DOCX)

S4 Table Mortality rates in the study group (AS patients) and in the control cohort. (DOCX)

S5 Table Annual health expenditures per capita in ankylosing spondylitis (AS). (DOCX)

S1 Text Extended guidelines: Definition of physical disability due to spinal lesion, according to the National Health Insurance Service in Korea. (DOCX)
