# Supplementary material for: Comorbidity, disability, and healthcare expenditure of ankylosing spondylitis in Korea: A population-based study
Source: PLoS One. 2018 Feb 8;13(2):e0192524. doi: 10.1371/journal.pone.0192524 (PMC5805317; doi:10.1371/journal.pone.0192524)

**S1 Fig.** Flow chart for selection of patients included in the final analysis. Of over one million individuals enrolled in the NHIS-NSC, we included 1,111 patients diagnosed with ankylosing spondylitis (AS) between 2003 and 2013. Age-, sex-, income-, and geographic region-matched controls were also selected from the NHIS-NSC.

NHIS-NSC, National Health Insurance Service-National Sample Cohort; RA, rheumatoid arthritis; SLE, systemic lupus erythematosus.


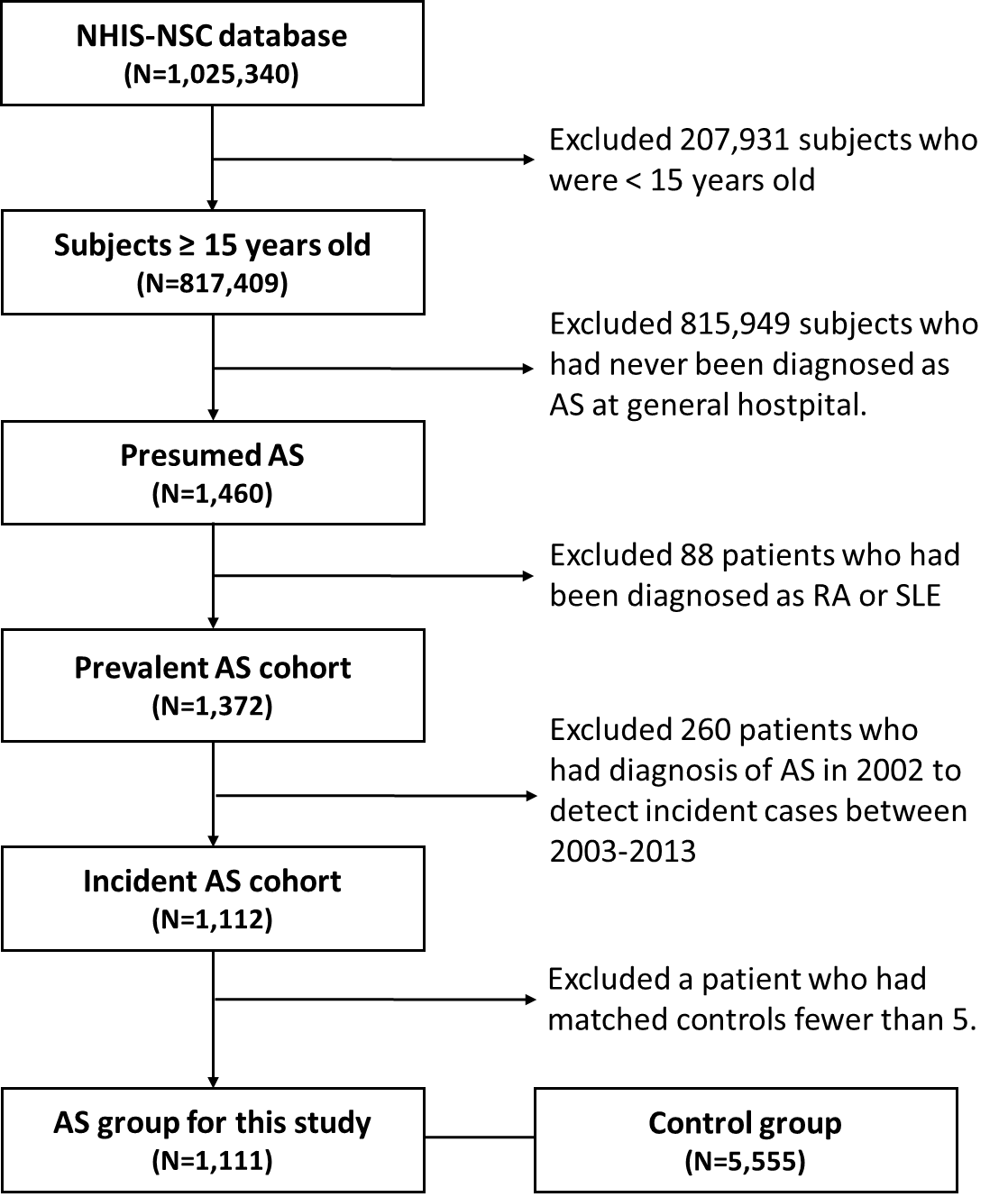

Supplement: S1 Fig — (DOCX) [file pone.0192524.s001.docx]
