# Supplementary material for: Comorbidity, disability, and healthcare expenditure of ankylosing spondylitis in Korea: A population-based study
Source: PLoS One. 2018 Feb 8;13(2):e0192524. doi: 10.1371/journal.pone.0192524 (PMC5805317; doi:10.1371/journal.pone.0192524)

**S2 Fig.** Forest plot presenting the result of multivariate logistic regression analysis for severe disability within the study group of patients with ankylosing spondylosis. (A) All-cause disability. (B) Physical disability.

95%CI, 95% confidence interval; CCI, Charlson comorbidity index; EAM, extra-articular manifestation; FU, follow-up; OR, odds ratio.


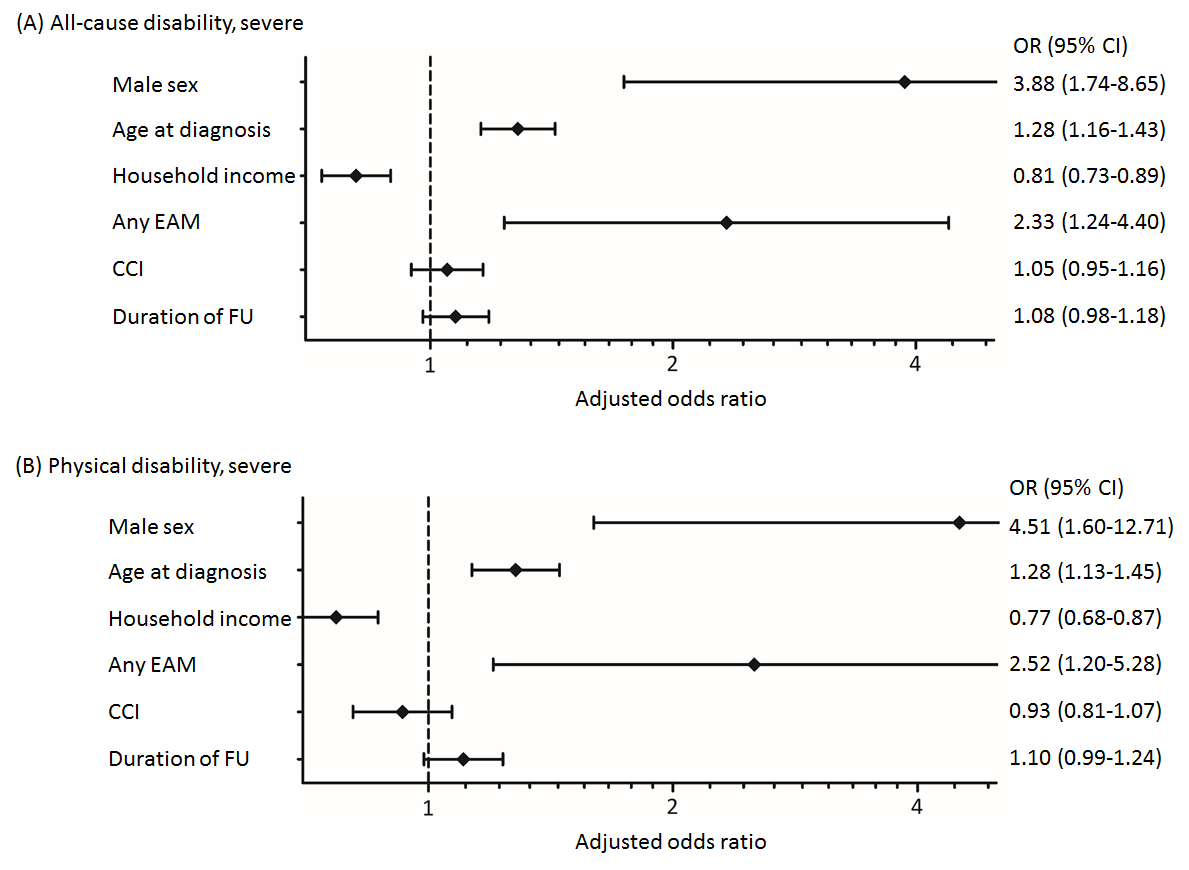

Supplement: S2 Fig — (DOCX) [file pone.0192524.s002.docx]
