# Supplementary material for: Comorbidity, disability, and healthcare expenditure of ankylosing spondylitis in Korea: A population-based study
Source: PLoS One. 2018 Feb 8;13(2):e0192524. doi: 10.1371/journal.pone.0192524 (PMC5805317; doi:10.1371/journal.pone.0192524)
Supplement: S1 Table — (DOCX) [file pone.0192524.s003.docx]

**S1 Table.** Stratification of demographic variables used for matching.

| **Variable** | **Strata** | **Variable** | **Strata** |
| --- | --- | --- | --- |
| Age group (4 strata) | 15-29 years | Geographic region | Seoul, metropolitan |
|  | 30-44 | (3 strata) | Large cities |
|  | 45-59 |  | Other areas |
|  | ≥60 |  |  |
| Income level (5 strata) | 1^st^ quintile (0-20%) | Sex (2 strata) | Male |
|  | 2^nd^ |  | Female |
|  | 3^rd^ |  |  |
|  | 4^th^ |  |  |
|  | 5^th^ (highest) |  |  |
|  |  |  |  |
|  |  |  |  |
