# Supplementary material for: Comorbidity, disability, and healthcare expenditure of ankylosing spondylitis in Korea: A population-based study
Source: PLoS One. 2018 Feb 8;13(2):e0192524. doi: 10.1371/journal.pone.0192524 (PMC5805317; doi:10.1371/journal.pone.0192524)
Supplement: S2 Table — (DOCX) [file pone.0192524.s004.docx]

**S2 Table.** Risk for overall comorbidity with a given Charlson comorbidity index (CCI) in ankylosing spondylitis

| Risk factor | | Odds ratio (95%CI) | | | | | | | | | | |
| --- | --- | --- | --- | --- | --- | --- | --- | --- | --- | --- | --- | --- |
|  | | CCI = 0 | | | CCI = 1 | | | CCI = 2 | | | CCI ≥ 3 | |
| All patients | 0.30 | | (0.23–0.38) | 0.60 | | (0.51–0.72) | 1.12 | | (0.95–1.32) | 2.18 | | (1.91–2.48) |
| Sex |  | |  |  | |  |  | |  |  | |  |
| Male | 0.34 | | (0.26–0.44) | 0.61 | | (0.50–0.74) | 1.28 | | (1.07–1.54) | 1.94 | | (1.69–2.24) |
| Female | 0.22 | | (0.12–0.41) | 0.68 | | (0.50–0.92) | 0.83 | | (0.63–1.09) | 1.48 | | (1.26–1.72) |
| Age at diagnosis |  | |  |  | |  |  | |  |  | |  |
| <45, years | 0.31 | | (0.24–0.40) | 0.66 | | (0.54–0.79) | 1.29 | | (1.08–1.55) | 2.38 | | (2.04–2.78) |
| ≥45, years | 0.33 | | (0.18–0.59) | 0.51 | | (0.35–0.76) | 0.75 | | (0.55-1.02) | 1.34 | | (1.17–1.55) |
| Household income |  | |  |  | |  |  | |  |  | |  |
| <4th quintile | 0.33 | | (0.24–0.45) | 0.75 | | (0.60–0.95) | 1.10 | | (0.88–1.37) | 1.71 | | (1.46–1.99) |
| ≥4th quintile, high | 0.32 | | (0.23–0.45) | 0.53 | | (0.42–0.68) | 1.12 | | (0.91–1.37) | 1.72 | | (1.50–1.98) |
| EAM |  | |  |  | |  |  | |  |  | |  |
| ≥1 | 2.37 | | (1.42–3.94) | 2.70 | | (1.80–4.04) | 5.91 | | (4.09–8.55) | 5.20 | | (4.23–6.39) |
| None | 0.22 | | (0.17–0.29) | 0.49 | | (0.40–0.59) | 0.81 | | (0.68–0.97) | 1.28 | | (1.12–1.46) |

Odds ratios with 95% confidence intervals (95%CIs) were computed taking the risk in the control cohort as reference. Controls were matched to the study group by age, sex, income, and geographic region.

EAM, extra-articular manifestation.
