# Supplementary material for: Comorbidity, disability, and healthcare expenditure of ankylosing spondylitis in Korea: A population-based study
Source: PLoS One. 2018 Feb 8;13(2):e0192524. doi: 10.1371/journal.pone.0192524 (PMC5805317; doi:10.1371/journal.pone.0192524)
Supplement: S3 Table — (DOCX) [file pone.0192524.s005.docx]

**S3 Table.** Crude incidence rates of disability in the study group (AS patients) and the control cohort

|  | Incidence rate*  AS patients (n = 1,111) | | | |  | Incidence rate*  Controls (n = 5,555) | | | |  |
| --- | --- | --- | --- | --- | --- | --- | --- | --- | --- | --- |
| Risk factor | All-cause | All-cause, severe | Physical | Physical, severe |  | All-cause | All-cause, severe | Physical | Physical, severe | |
| All patients | 32.46 | 7.52 | 26.54 | 5.28 |  | 11.04 | 3.54 | 4.98 | 0.46 | |
| Sex |  |  |  |  |  |  |  |  |  | |
| Male | 40.45 | 9.98 | 34.15 | 7.35 |  | 11.71 | 4.01 | 5.03 | 0.55 | |
| Female | 20.02 | 3.68 | 14.71 | 2.04 |  | 9.94 | 2.75 | 4.89 | 0.31 | |
| Age at diagnosis |  |  |  |  |  |  |  |  |  | |
| <45 years | 17.94 | 3.69 | 15.49 | 3.20 |  | 6.80 | 3.01 | 2.87 | 0.37 | |
| ≥45 years | 59.47 | 14.64 | 47.12 | 9.15 |  | 18.04 | 4.41 | 8.45 | 0.61 | |
| Household income |  |  |  |  |  |  |  |  |  | |
| <4th quintile | 39.81 | 10.79 | 32.74 | 8.18 |  | 14.22 | 5.15 | 5.81 | 0.59 | |
| ≥4th quintile, high | 26.92 | 5.05 | 21.87 | 3.08 |  | 8.54 | 2.26 | 4.32 | 0.36 | |
| Duration of follow-up |  |  |  |  |  |  |  |  |  | |
| <5 years | 188.74 | 44.47 | 154.15 | 31.62 |  | 69.42 | 25.26 | 30.76 | 3.30 | |
| ≥5 years | 2.29 | 0.38 | 1.91 | 0.19 |  | 2.25 | 0.26 | 1.09 | 0.03 | |
| EAM |  |  |  |  |  |  |  |  |  | |
| ≥1 | 46.12 | 12.81 | 36.52 | 9.61 |  | 13.23 | 3.61 | 4.01 | 0.80 | |
| None | 27.91 | 5.75 | 23.23 | 3.84 |  | 10.88 | 3.53 | 5.05 | 0.43 | |
| Comorbidity |  |  |  |  |  |  |  |  |  | |
| CCI ≥ 3 | 39.50 | 9.46 | 30.60 | 6.40 |  | 18.04 | 4.79 | 8.46 | 0.60 | |
| CCI = 2 | 25.74 | 5.46 | 23.40 | 3.90 |  | 7.40 | 2.27 | 3.47 | 0.30 | |
| CCI = 1 | 17.95 | 5.98 | 16.95 | 4.99 |  | 6.96 | 2.66 | 3.29 | 0.51 | |
| CCI = 0 | 26.74 | 0 | 24.06 | 0 |  | 5.67 | 3.34 | 1.60 | 0.29 | |

Controls were matched to the study group by age, sex, income, and geographic region.

*per 1000 person-years

AS, ankylosing spondylitis; CCI, Charlson comorbidity index; EAM, extra-articular manifestation.
