# Supplementary material for: Comorbidity, disability, and healthcare expenditure of ankylosing spondylitis in Korea: A population-based study
Source: PLoS One. 2018 Feb 8;13(2):e0192524. doi: 10.1371/journal.pone.0192524 (PMC5805317; doi:10.1371/journal.pone.0192524)
Supplement: S4 Table — (DOCX) [file pone.0192524.s006.docx]

**S4 Table.** Mortality rates in the study group (AS patients) and in the control cohort

| Risk factor | | Incidence rate*  AS patients (n = 1,111) | | | Incidence rate*  Controls (n = 5,555) | | Incidence rate ratio (95%CI) | |
| --- | --- | --- | --- | --- | --- | --- | --- | --- |
| All patients |  | | 7.18 |  | | 5.90 | 1.22 | (0.90–1.65) |
| Sex |  | |  |  | |  |  |  |
| Male |  | | 7.06 |  | | 5.40 | 1.31 | (0.89–1.93) |
| Female |  | | 7.38 |  | | 6.73 | 1.10 | (0.68–1.78) |
| Age at diagnosis |  | |  |  | |  |  |  |
| <45 years |  | | 1.34 |  | | 1.39 | 0.97 | (0.40–2.31) |
| ≥45 years |  | | 16.61 |  | | 13.01 | 1.28 | (0.92–1.76) |
| Household income |  | |  |  | |  |  |  |
| <4th quintile |  | | 8.09 |  | | 6.65 | 1.22 | (0.79–1.87) |
| ≥4th quintile, high |  | | 6.45 |  | | 5.30 | 1.22 | (0.79–1.87) |
| Duration of follow-up |  | |  |  | |  |  |  |
| <5 years |  | | 29.87 |  | | 26.29 | 1.14 | (0.75–1.73) |
| ≥5 years |  | | 3.94 |  | | 3.06 | 1.29 | (0.83–2.00) |
| EAM |  | |  |  | |  |  |  |
| ≥1 |  | | 5.82 |  | | 6.11 | 0.95 | (0.44–2.05) |
| None |  | | 7.66 |  | | 5.88 | 1.30 | (0.93–1.82) |
| Comorbidity |  | |  |  | |  |  |  |
| CCI ≥ 3 |  | | 10.23 |  | | 11.59 | 0.88 | (0.63–1.23) |
| CCI = 2 |  | | 3.48 |  | | 3.21 | 1.08 | (0.41–2.86) |
| CCI = 1 |  | | 0.91 |  | | 1.23 | 0.75 | (0.10–5.82) |
| CCI = 0 |  | | 4.89 |  | | 2.27 | 2.15 | (0.50–9.37) |
| Disability |  | |  |  | |  |  |  |
| All-cause |  | | 16.45 |  | | 15.72 | 1.05 | (0.62–1.76) |
| All-cause, severe |  | | 19.74 |  | | 27.25 | 0.72 | (0.29–1.80) |
| Physical |  | | 16.29 |  | | 7.53 | 2.16 | (0.97–4.82) |
| Physical, severe |  | | 14.02 |  | | 21.28 | 0.66 | (0.11–3.94) |

Controls were matched to the study group by age, sex, income, and geographic region.

*per 1000 person-years

95%CI, 95% confidence interval; AS, ankylosing spondylitis; CCI, Charlson comorbidity index; EAM, extra-articular manifestation.
