# Supplementary material for: Comorbidity, disability, and healthcare expenditure of ankylosing spondylitis in Korea: A population-based study
Source: PLoS One. 2018 Feb 8;13(2):e0192524. doi: 10.1371/journal.pone.0192524 (PMC5805317; doi:10.1371/journal.pone.0192524)
Supplement: S5 Table — (DOCX) [file pone.0192524.s007.docx]

**S5 Table.** Annual health expenditures per capita in ankylosing spondylitis (AS)

|  | AS patients (n=1111) | | AS patients treated with biologics (n=128) | | Controls (n=5555) | |
| --- | --- | --- | --- | --- | --- | --- |
|  | Median | (IQR) | Median | (IQR) | Median | (IQR) |
| All patients | 596 | (295–1840) | 4549 | (2729–6348) | 183 | (62–494) |
| Sex |  |  |  |  |  |  |
| Male | 552 | (262–2004) | 4542 | (2650–5974) | 135 | (45–372) |
| Female | 677 | (358–1504) | 5216 | (3856–8071) | 288 | (118–680) |
| Age at diagnosis |  |  |  |  |  |  |
| <45 years | 459 | (238–1032) | 4400 | (2566–5570) | 112 | (41–276) |
| ≥45 years | 1063 | (451–2713) | 5897 | (3213–7924) | 408 | (156–1034) |
| Household income |  |  |  |  |  |  |
| <4th quintile | 589 | (261–2098) | 4547 | (2741–6347) | 160 | (54–466) |
| ≥4th quintile, high | 608 | (314–1575) | 4552 | (2714–6577) | 193 | (71–524) |
| Duration of follow-up |  |  |  |  |  |  |
| <5 years | 765 | (397–2152) | 5188 | (3877–7072) | 151 | (49–475) |
| ≥5 years | 501 | (244–1599) | 3760 | (2274–5638) | 198 | (71–511) |
| EAM |  |  |  |  |  |  |
| ≥1 | 864 | (400–2711) | 4542 | (2767–6742) | 344 | (141–937) |
| None | 534 | (259–1494) | 4558 | (2688–5829) | 172 | (58–472) |
| Comorbidity |  |  |  |  |  |  |
| CCI≥3 | 940 | (419–2502) | 4718 | (3164–6978) | 477 | (214–1043) |
| CCI=2 | 453 | (242–984) | 3209 | (1943–5061) | 188 | (88–359) |
| CCI=1 | 310 | (140–681) | 4846 | (3153–6209) | 93 | (40–218) |
| CCI=0 | 307 | (152–555) | 4663 | (2669–5111) | 41 | (13–113) |
| Disability |  |  |  |  |  |  |
| All-cause | 1987 | (577–3942) | 5210 | (2666–6995) | 698 | (210–1791) |
| All-cause, severe | 2741 | (1106–6514) | 6871 | (6091–7943) | 558 | (127–2639) |
| Physical | 1951 | (520–3457) | 4551 | (2629–6766) | 673 | (206–1548) |
| Physical, severe | 2371 | (581–6409) | 6409 | (5854–6929) | 385 | (112–1002) |
| Mortality |  |  |  |  |  |  |
| Yes | 2865 | (967–4412) |  | - | 1427 | (401–3296) |
| No | 560 | (282–1589) |  | - | 173 | (60–458) |

The calculations were performed for the period between 2003 and 2013. The original calculation was performed in KRW, and the final result was converted to USD at the rate of 1 USD = 1100 KRW.

IQR, interquartile range 25^th^–75^th^ percentiles;
