# Supplementary material for: Comorbidity, disability, and healthcare expenditure of ankylosing spondylitis in Korea: A population-based study
Source: PLoS One. 2018 Feb 8;13(2):e0192524. doi: 10.1371/journal.pone.0192524 (PMC5805317; doi:10.1371/journal.pone.0192524)
Supplement: S1 Text — (DOCX) [file pone.0192524.s008.docx]

**S1 Text.** Extended guidelines: Definition of physical disability due to spinal lesion, according to the National Health Insurance Service in Korea

A. Defining spinal disability

A1. The spinal lesion should be evaluated by plain X-ray, computed tomography, magnetic resonance imaging, and electromyography. The type and location of the lesion should be determined, as well as signs of previous surgery.

A2. Segments treated via artificial disc insertion, dynamic fixation, or wire fixation to achieve spinal movement are not regarded as fixed segments.

A3. In patients with ankylosing spondylitis (AS), radiographic evidence of spinal lesion should be definite. Sacroiliac joint lesions are not considered. Disabilities in the upper and lower spine are evaluated separately. Complete ankylosis is defined as complete fusion of cervical, thoracic, or lumbar spine segments, with 90% decrease in range of motion.

A4. According to the spinal level involved, normal range of motion is defined differently, as listed below.

| Cervical | Occiput-C1 | C1-2 | C2-3 | C3-4 | C4-5 | C5-6 | C6-7 | C7-T1 | Sum |
| --- | --- | --- | --- | --- | --- | --- | --- | --- | --- |
| Range of motion, ° | 13 | 10 | 8 | 13 | 12 | 17 | 16 | 6 | 95 |
| Thoracolumbar | T10-11 | T11-12 | T12-L1 | L1-2 | L2-3 | L3-4 | L4-5 | L5-S1 | Sum |
| Range of motion, ° | 9 | 12 | 12 | 12 | 14 | 15 | 17 | 20 | 111 |

B. Defining the severity of spinal disability

| Disability grade | Description |
| --- | --- |
| Grade 2-5 | Range of motion in the cervical and thoracolumbar spine decreased by 80% |
| Grade 2-6 | AS-related complete ankylosis of the cervical and thoracolumbar spine |
| Grade 3-1 | Range of motion in the cervical or thoracolumbar spine decreased by 80% |
| Grade 4-1 | Range of motion in the cervical or thoracolumbar spine decreased by 60% |
| Grade 5-8 | Range of motion in the cervical or thoracolumbar spine decreased by 40% |
| Grade 5-9 | AS-related complete ankylosis of the cervical and thoracic spine or of the thoracolumbar spine |
| Grade 6-5 | Range of motion in the cervical or thoracolumbar spine decreased by 20% |
| Grade 6-6 | AS-related complete ankylosis of the cervical or lumbar spine |
